# Initiation of Chemosaturation With Percutaneous Hepatic Perfusion Program in Interventional Radiology Department

**DOI:** 10.7759/cureus.17880

**Published:** 2021-09-10

**Authors:** Osman Öcal, Gonca Eldem, Ayse H Karagoz, Saadettin Kılıçkap, Suayib Yalcin, Ferhun Balkanci, Bora Peynircioglu

**Affiliations:** 1 Radiology, Hacettepe University, Ankara, TUR; 2 Anesthesiology and Reanimation, Hacettepe University, Ankara, TUR; 3 Medical Oncology, Hacettepe University, Ankara, TUR

**Keywords:** colorectal cancer, melanoma, liver, percutaneous hepatic perfusion, chemosaturation

## Abstract

Objectives

Chemosaturation with percutaneous hepatic perfusion (PHP) is a relatively new minimally-invasive liver-directed therapy, which aims to deliver high-dose chemotherapy into the liver with low systemic side effects. Initial studies showed promising results, especially in the treatment of metastatic uveal melanoma. But unfamiliarity of the interventional radiologists prevents its widespread implantation in clinical routine. This study aimed to outline how to initiate a PHP program and report initial results.

Methods

We retrospectively reviewed all patients who underwent chemosaturation with PHP in our institution between March 2016 and February 2017 and their follow-up results till October 2018. Patient demographics, procedural characteristics, clinical and imaging results, and complications were evaluated. Additionally, modifications regarding infrastructure and procedure techniques were described.

Results

A total of three patients (two females and one male) with a mean age of 59 underwent six PHP procedures. The primary disease was colorectal carcinoma in one patient and uveal melanoma in two patients. The technical success rate was 100% and the mean melphalan dose was 190.8 mg. No procedural death was observed. Patients were hospitalized for a mean of 3.3 days after procedures. Grade 3 and 4 complications were seen after 50% and 33.3% of procedures, respectively. Two patients showed partial response and the other patient showed stable disease after procedures. Mean hepatic progression-free survival was 10.8 months. Overall survival from the first procedure was 14.8 months in our cohort.

Conclusion

Our results show that chemosaturation with PHP offers a promising minimally invasive treatment option in patients with unresectable liver metastases. The technical challenges of PHP can be easily handled by an experienced interventional radiology (IR) team. It is a relatively safe procedure and its toxicities are usually hematological and can be manageable with close surveillance and appropriate medical therapies.

## Introduction

Chemosaturation with percutaneous hepatic perfusion (PHP) is a minimally invasive procedure for infusion of high doses of melphalan into hepatic circulation with limited systemic exposure. Hepatic veins are isolated from systemic circulation with a percutaneously placed dedicated double-balloon catheter before the infusion of melphalan into the hepatic artery, and infused drug filtrated through an extracorporeal circuit under venovenous bypass. PHP has been shown to be effective in the treatment of various primary and secondary liver malignancies [1-4]. A randomized prospective study of PHP with melphalan in melanoma patients showed a significant increase in hepatic disease control compared to best alternative care with minimal systemic and hepatic toxicity [3]. But the procedure requires to exit the daily clinical routine of interventional radiology (IR), such as implantation of an extracorporeal bypass machine in the angiography suite and working with a perfusionist during the procedure. This limits the widespread implantation of the technique. According to the manufacturer of the double-balloon catheter, currently, PHP is done in only 16 centers in six countries. This report outlines the medical infrastructure and technical considerations that are required to initiate a PHP program and shows our preliminary results with the first three cases.

## Materials and methods

This retrospective study was approved by the ethics committee of our university hospital under number GO 17/46-20 and conducted following the Helsinki declaration. We reviewed the prospectively collected database for all the patients who underwent PHP in our interventional radiology unit between March 2016 and February 2017 and their follow-up results until their death. Patient and disease characteristics, procedure details, clinical and radiological follow-up results, and procedure-related complications were collected. The treatment response of the hepatic lesions was assessed using RECIST (Response Evaluation Criteria in Solid Tumors) v1.1. Any grade 3 or 4 adverse events according to CTCAE (Common Terminology Criteria for Adverse Events) version 5.0 were recorded up to 30 days after each procedure. Additionally, challenges that were encountered regarding infrastructure, organization of daily workflow, and peri-procedural technical details, and our team effort in solving them were described.

Pre-procedural assessments

All patients were referred from the oncology department of our institution and underwent physical examination, laboratory tests, and echocardiography to evaluate the eligibility of the patients for the procedure (Table 1). Upper abdominal magnetic resonance imaging (MRI) with hepatospecific contrast medium was done to evaluate hepatic disease burden and the presence of extrahepatic disease was assessed with positron emission tomography (PET). Brain MRI and bone scintigraphy were utilized if clinically indicated.

**Table 1:** Patient selection criteria

Inclusion Criteria	
Presence of unresectable hepatic metastases	
Limited extrahepatic disease	
Age	>18 years
Bodyweight	>35 kg
ECOG	<2
Bilirubin	<3 mg/dl
INR	<2 s
ALT/AST	< ×10 normal
Absolute neutrophil count	>1,300
Platelet count	>75,000/dl
Hemoglobin	>9 g/dl
Adequate renal function (GFR)	>60 ml/min/1.73 m
Exclusion Criteria	
Contraindication to general anesthesia	
Heart failure (ejection fraction)	<40%
Portal hypertension (ascites, recent variceal hemorrhage, or encephalopathy)	
Melphalan or heparin hypersensitivity	
Iodine allergy	
History of gastrinoma or Whipple procedure	
Active infection	

Antihypertensive medications such as angiotensin-converting enzyme inhibitors, angiotensin receptor inhibitors, calcium channel blockers were altered before the treatment. Four units of erythrocyte, four units of fresh frozen plasma, and ten units of thrombocytes and cryoprecipitates were prepared before every procedure. Protein pump inhibitors and 300 mg allopurinol were started three days prior to treatments. The total dose of melphalan was calculated by the Oncologist as 3 mg/kg and corrected for ideal body weight. All patients were hospitalized one day prior to treatment and hydrated appropriately for 12 hours before the procedure and antibiotic prophylaxis was done if there were any history of previous hepatobiliary surgery. Any anticoagulation drug was ceased before the procedure, according to instructions.

Procedural details

All PHP procedures were performed in the IR suite under general anesthesia. The procedure team consisted of interventional radiologists, anesthesiologists, and one perfusionist. Two IR rooms were designated; one for the PHP procedure and one to assemble and prime the hemofiltration circuit (GEN2 filter; Delcath Systems, Inc., New York, USA), hydrate the chemofilters, and test the pressure of the circuit, which was done by the perfusionist. The radial artery was cannulated for invasive blood pressure monitoring and a central venous catheter was placed into the left jugular vein for hydration and infusion of anesthetic or vasopressor drugs. The patients were heated with air blankets throughout the procedure.

**Figure 1:**
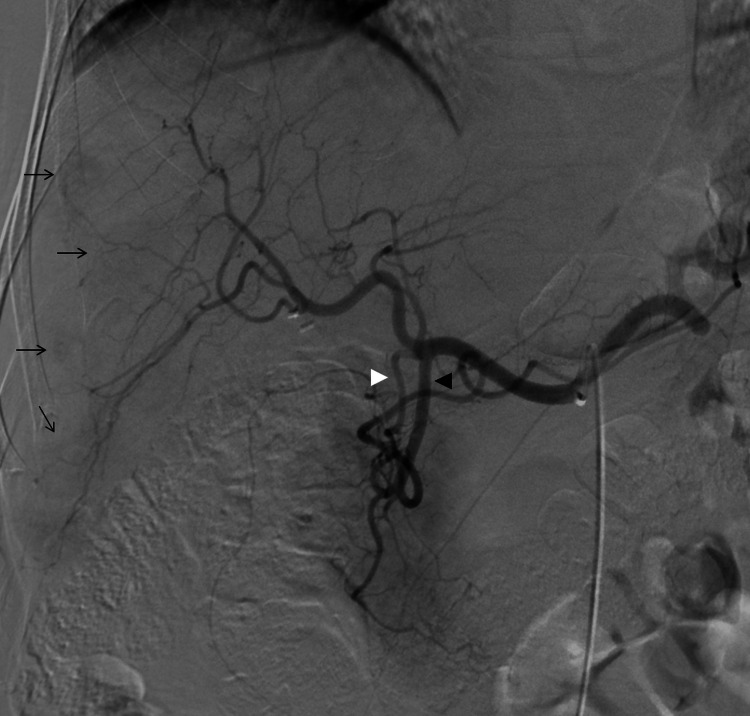
Procedural planning Celiac angiography shows gastroduodenal (black arrowhead) and supraduodenal artery (white arrowhead) originating from the common hepatic artery. A selective lobar infusion was decided. Multiple hypervascular metastases are also seen (arrows).

After induction of anesthesia, a 5 F introducer sheath was placed into the left femoral artery. This was followed by the placement of 18 F and 10 F venous sheaths into the right femoral and internal jugular vein, respectively. All punctures were made under ultrasonography guidance to avoid double-wall penetration. After completion of vascular access, systemic anticoagulation with heparin was started to achieve activated clotting time (ACT) greater than 400 seconds. Celiac and superior mesenteric angiograms were obtained to delineate hepatic vascular anatomy and to check for any possible extrahepatic supply (Figure 1).

**Figure 2:**
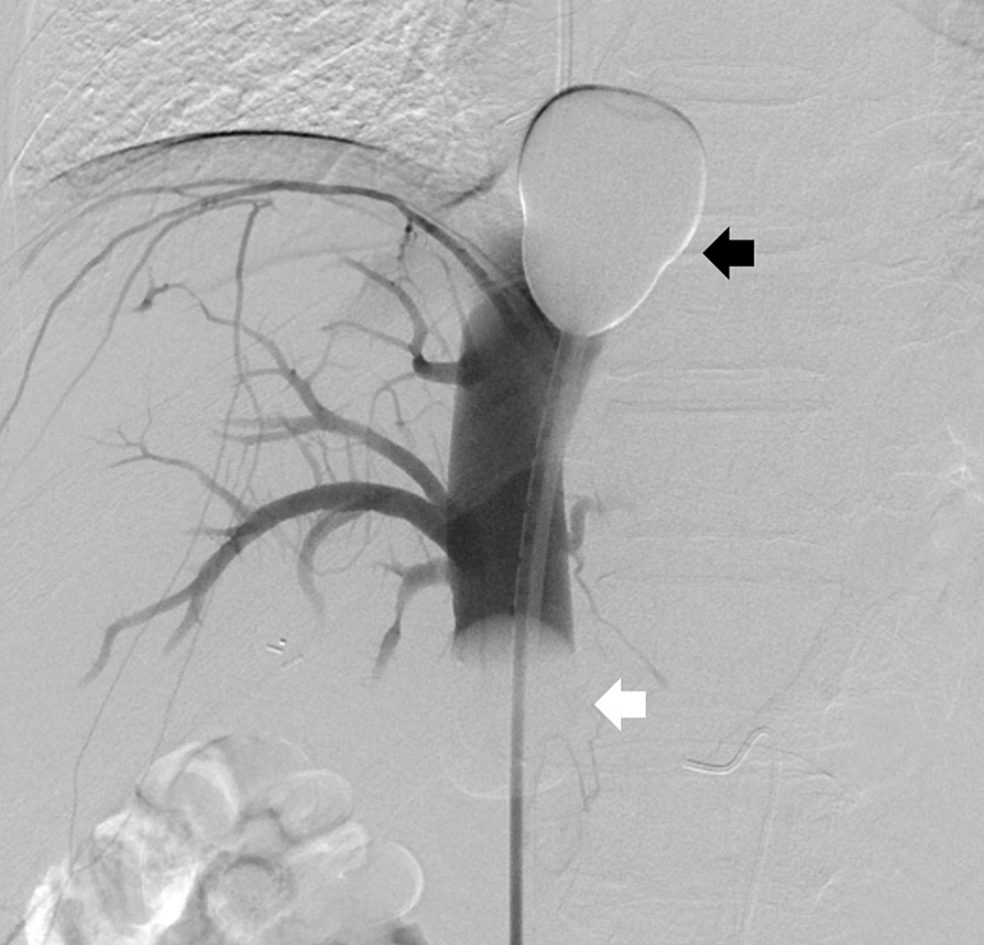
Venography Venography via fenestrations of the double-balloon catheter shows complete isolation of the hepatic venous outflow. Cranial balloon (black arrow) seals cavo-atrial junction and caudal balloon (white arrowhead) is in infrahepatic inferior vena cava.

A microcatheter (2.4-2.8 F) was positioned into the artery at the intended location of melphalan infusion. After that, the 16 F double-balloon catheter (Isofuse Catheter, Hepatic CHEMOSAT Delivery System, Delcath Systems, Inc., Queensbury, New York, USA) was placed into IVC through the right femoral venous sheath, positioning the cranial end in the right atrium. The cephalad balloon was then inflated with 50% contrast and retracted until seals the cavoatrial junction to stop venous return from IVC to the right atrium. Then the caudal balloon was inflated with 10% contrast to isolate hepatic veins completely from systemic circulation. Isolation of the hepatic venous outflow was controlled by angiography through fenestrations of the catheter between two balloons (Figure 2).

If any leakage was encountered, balloons were deflated and repositioned. After all the connections were checked and isolation of the hepatic veins were confirmed, extracorporeal circulation was started. Venous blood is aspirated from the large lumen through the fenestrations in the double-balloon catheter. This blood flows to the pump and through the bypass line and returns to the patient through the venous return sheath placed in the right jugular vein. Then the cephalad balloon was then inflated with 50% contrast and retracted until it seals the cavoatrial junction to stop venous return from IVC to the right atrium. Then the caudal balloon was inflated with 10% contrast to isolate the hepatic veins completely from the systemic circulation. Isolation of the hepatic venous outflow was controlled by angiography through fenestrations of the catheter between two balloons. If any leakage was encountered, balloons were deflated and repositioned. After a satisfactory position isolating the hepatic veins was attained, gentle constant back pressure holding the hub of the double-balloon catheter was done to prevent upward migration. Then the extracorporeal circulation was directed to the hemofiltration filters. This time venous outflow of the liver flows through the fenestrations of the catheter between two balloons and pumped through the filters and after filtration blood returns to the systemic circulation via the catheter in the right internal jugular vein. After every step in establishing the extracorporeal circulation and filtration, serious hypotension with systolic pressures as low as 45 mmHg occurs, and aggressive hydration and vasopressor support with norepinephrine is required with the support of the anesthesiology team until the patient is stabilized. After stabilization of the patient, the calculated total dose of melphalan diluted into 500 cc saline was infused through the arterial microcatheter using the angiographic injector with a rate of 0.4 mL/sec. Hepatic angiograms were obtained to check the microcatheter position and the presence of vasospasm during the infusion period. If vasospasm occurred, it was relieved with nitroglycerine infusion (100 µg). After chemotherapeutic infusion was completed, an additional 30-minute washout period was waited to clear up any residual melphalan within the liver.

Once the procedure was completed, heparin was reversed with protamine sulfate and hemostasis was achieved with an Angioseal closure device on the arterial access site. Patients were then transferred to the intensive care unit (ICU) and femoral and jugular venous vascular sheaths were removed and hemostasis was achieved with manual compression when all coagulation parameters returned to normal levels. Blood products were transfused as needed to replace clotting factors.

Follow-up

All patients were followed in the intensive care unit for 12-24 hours and hospitalized for two to three days. Complete blood counts, liver and kidney function tests, and coagulation parameters were checked daily during hospitalization, and then at three-day intervals for two weeks after discharge. Follow-up imaging was done every six weeks during the active treatment period. After the active treatment period, patients were seen in the clinic monthly and imaged bimonthly with liver MRI. Extrahepatic tumor burden was evaluated with thorax CT, brain MRI, or bone scintigraphy when clinically indicated. Technical challenges of PHP and strategies to overcome these were summarized in Table 2.

**Table 2:** Pre-, peri- and post-procedural challenges and strategies to modify and accelerate solutions A: anesthesiologist, ACEI: angiotensin-converting enzyme inhibitors, ARB: angiotensin receptor blockers, CCB: Ca channel blockers, ICU: intensive care unit, IR: interventional radiologist, MO: medical oncologist, P: perfusionist.

Pre-Work Up	Team Members	Encountered Difficulty	Modifications
Evaluation of patient eligibility	MO, IR	None	Please see Table 1
Evaluation for the procedure	IR, A	None	none
Melphalan dose calculation	MO	None	According to ideal body weight
Peri-procedural workup	Team members	Encountered difficulty	Modifications
Preparation of blood products	IR	Excessive need	Pre-order from blood bank: 4 units erythrocytes, 4 units fresh frozen plasma, 10 units thrombocytes, 10 units cryoprecipitates
Daily workflow	IR	Long duration of PHP	All daily elective cases postponed
Circuit assembly	P	Need for at least 45 min-1 hour to assemble	Designate another IR room to accelerate
Circuit assembly; priming and hydration	P	De-bubble all the lines, circuits, and filters with saline	Doing it with CO_^2^_ is faster
General anesthesia induction heated saline heating blankets temperature measurement	A	Not routine equipment used in daily IR suites	Provided from operating rooms
Procedure	Team Members	Encountered Difficulty	Modifications
Arterial and venous sheath insertion	IR	None	US guidance to avoid double-wall puncture
Celiac angiography and positioning the catheter	IR	None	Co-axial catheterization with 4 F Sim and 2.8 F microcatheter
Positioning the double-balloon catheter	IR	None	Venography to exclude leakage
Extracorporeal circulation	P	None	None
Extracorporeal filtration	P	None	None
Managing hypotension during extracorporeal circulation and filtration Vasopressor test with phenylephrine	A	The vasopressor test was not done as phenylephrine is not provided in our country	Norepinephrine was used as a vasopressor. ACEI, ARB, and CCBs were bridged before the procedure.
Post-procedure	Team members	Encountered difficulty	Modifications
Early adverse events	A, IR	None	ICU follow-up
Late adverse events	O, IR	Hematological toxicities	Colony-stimulating factors were started immediately

Results of the continuous data are displayed as means and standard deviations, results of frequency data as counts and percentages. The statistical analysis was performed using R statistical software (v.3.6.3; R Foundation for Statistical Computing, Vienna, Austria).

## Results

**Figure 3:**
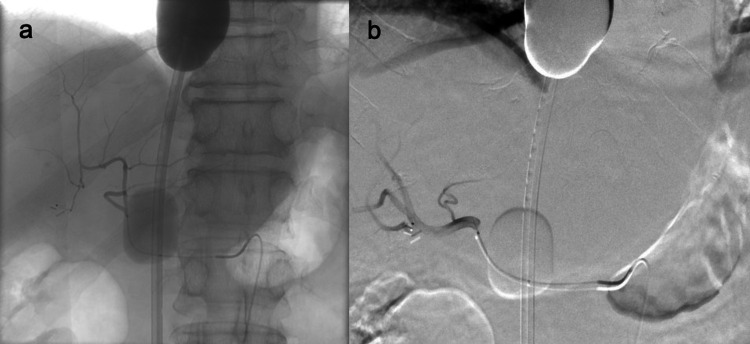
Melphalan infusion Angiographic image of the (a) left hepatic and (b) right hepatic artery show microcatheter position for melphalan infusion.

Three patients (two females with ocular melanoma and one male with colorectal carcinoma) with a mean age of 59 (range,54-64 years) were identified, all were metastasis-free at the initial diagnosis. Later, they presented with diffuse hepatic metastases (>10 lesions), which progressed despite multiple local and systemic treatments, including radioembolization. Except for the patient with stable bone metastasis, all patients had the liver-only disease before the first PHP procedure. Each patient underwent two procedures. Technical success was 100%, and administration of the pre-planned melphalan dose was successful in all procedures with a mean melphalan dose of 190.8 mg (range, 169-220 mg). The mean procedural time was 119 (range, 105-131) minutes, and the mean total melphalan infusion time was 29.8 (range, 21-39) minutes. In two patients, the infusion catheter was positioned in the proper HA. In the other patient, 60% of the dose was infused into the right HA, and the rest into the left HA (Figure 3).

All patients spent one night in the intensive care unit and remained in the hospital for two to five days (mean 3.3 days) after the PHP procedure. The interval between procedures ranged from 12 to 16 weeks. Patient demographics and procedure characteristics were summarized in Table 3.

**Table 3:** Patient and procedure characteristics ES: erythrocyte suspension, F: female, FFP: fresh frozen plasma, M: male, mo: months, OS: overall survival, Plt: platelets, PFS: progression-free survival.

Patient	Age/gender	Disease	Time to liver lesions	No. of PHP	Melphalan dose (mg)	Procedure and infusion duration (minutes)	In-hospital transfusions	Late toxicities (discharge - 30 days)	Postdischarge transfusions	Hepatic PFS	OS (since 1^st ^PHP)	OS (since diagnosis)
1	54/F	UM	36 mo	2	168	120/39	3 ES, 2 FFP, 2 aphereses	No grade 3 toxicity	-	7.2 mo	9.8 mo	62 mo
					170	121/28	2 ES, 2 FFP, 6 Plt	G3 anemia, G4 thrombocytopenia	1 ES, 4 Plt			
2	59/F	UM	42 mo	2	198	105/21	2 Aphereses, 2 FFP	No grade 3 toxicity	-	7.9 mo	10.5 mo	69 mo
					169	131/25	1 Apheresis, 4 cryoprecipitate, 1 FFP		-			
3	64/M	CRC	2 mo	2	R (130) and L (90)	115/31	2 ES, 2 FFP, 3 aphereses	G3 anemia, G3 thrombocytopenia	-	17.5 mo	24.2 mo	53 mo
					R (175) and L (45)	122/35	2 ES, 3 aphereses, 5 FFP	G3 anemia, G4 thrombocytopenia, G4 neutropenia, G3 hyperbilirubinemia	1 ES, 15 Plt			

No adverse events of grade ≥3 were encountered during the procedures. One patient had transient arrhythmias related to over-retraction of the double-balloon catheter, which was relieved after deflation of balloons at the end of the procedure. None of the patients had complications related to vascular access. Due to sequestration at the filters and hemodilution, patients received a mean of 1.5 units of packed red blood cells, two units of platelets, 1.1 units of fresh frozen plasma, and 0.7 units of cryoprecipitate during hospitalization.

After discharge, grade 3 and 4 hematologic toxicities were seen after 50% and 33.3% of procedures, respectively. No grade 4 hepatotoxicity was encountered. Two patients were hospitalized two weeks after the second procedure due to grade 4 hematologic toxicity and received a total of 19 units of platelets and two units of packed red blood cells. Patients were hospitalized until normalization of blood counts (one and nine days).

Follow-up imaging revealed a partial response in two patients and stable disease in one patient. The mean hepatic progression-free survival after the first PHP session was 10.8 months (range 7.2, 7.9, 17.5 months). All patients were lost due to disease progression and the mean overall survival after the first PHP procedure was 14.8 months (9.8, 10.5, and 24.2). Mean survival from the diagnosis of the primary tumor and hepatic metastases were 61.3 and 38.6 months.

## Discussion

PHP is a relatively new minimally invasive procedure for the infusion of high-dose melphalan into the liver. Hepatic venous outflow is isolated from systemic circulation with a percutaneously placed double-balloon catheter and directed to extracorporeal filters to minimize systemic exposure of melphalan. The initial phase I study was done in 1994 and showed filters eliminate 85.6% of the chemotherapeutic agent [4], which is improved to 98% with the development of second-generation filters [5]. Another phase I study performed in the same year showed an objective response rate of 9.5% [6]. A few studies and case reports showed that PHP is effective in various primary and secondary liver malignancies [1-3,7-10]. A randomized prospective study showed PHP improves hepatic disease control in patients with hepatic metastases from melanoma compared to best alternative care [1]. PHP showed improved hepatic and overall progression-free survival than both chemoembolization and radioembolization in patients with liver metastasis of uveal melanoma [11]. Despite the promising results and long history of PHP, it is still done only in a number of centers. PHP has some unique technical aspects compared to routine IR procedures. Isolation of the liver vasculature with a double-balloon catheter has some challenges for both interventionalist and anesthesiologists. Precise positioning of the balloons and accurate evaluation of any leakage in venography is crucial for minimizing systemic melphalan exposure. Veno-venous bypass requires systemic heparinization (ACT>400), and vascular access complications may cause significant hemorrhages. Also, early recognition of arterial spasm is crucial with intermittent angiographies to prevent non-target infusion. However, these challenging steps can be handled by an experienced interventional radiologist. Although an IR instructor did not accompany any of the procedures, an experienced anesthesiology instructor, together with a very experienced instructor for perfusionist, was present in all three patients. No procedure-related serious complication or mortality was encountered in our experience.

Patients must be followed for toxicity in the early post-procedural period. The most common adverse events were late leukopenia and thrombocytopenia in our patients due to incomplete removal of melphalan by filters, as in the literature [2]. Despite colony-stimulating factors, the cytopenia-related infectious complication was seen in one patient, and we recommend colony-stimulating factors as other authors [8,10]. All periprocedural complications were recognized early with intense follow-up in our cases and handled with appropriate medical care. None of the patients were lost due to complications. Mean hepatic progression-free and overall survivals after the first procedure were 10.8 and 14.8 months in this cohort, and it was similar with previous literature in PHP [1]. Considering all patients had progression after all alternative therapies, this overall survival is more promising for further patients.

This study has several limitations. This retrospective study was based on a small number of patients who had various therapies before and after the procedures. However, it aimed to present real-world oncology practice, and our results suggest that despite these challenges, with an experienced IR team, high success can be achieved even in the initial cases.

## Conclusions

In conclusion, PHP is a valuable treatment option in patients with unresectable liver metastases. Our results show PHP is a relatively safe procedure and can be repeated with limited toxicities. It offers prolonged survival for patients with hepatic metastases, even after all treatment options have failed. PHP can be established with high technical success and clinical efficacy in centers with an experienced multi-disciplinary team. Toxicities related to PHP are limited and can be detected early with intensive peri-procedural follow-up and handled with medical care.
